# Druggable chemical space and enumerative combinatorics

**DOI:** 10.1186/1758-2946-5-19

**Published:** 2013-04-18

**Authors:** Melvin J Yu

**Affiliations:** 1 Eisai Inc., 4 Corporate, Dr. Andover, MA, 01810, USA

**Keywords:** High-throughput screening, HTS, Combinatorics, Drug discovery, Drug space, Enumeration, Virtual compound libraries

## Abstract

**Background:**

There is a growing body of literature describing the properties of marketed drugs, the concept of drug-likeness and the vastness of chemical space. In that context, enumerative combinatorics with simple atomic components may be useful in the conception and design of structurally novel compounds for expanding and enhancing high-throughput screening (HTS) libraries.

**Results:**

A random combination of mono- and diatomic carbon, hydrogen, nitrogen, and oxygen containing components in the absence of molecular weight constraints but with the ability to form rings affords virtual compounds that fall in bulk physicochemical space typically associated with drugs, but whose ring assemblies fall in new or under-represented areas of chemical shape space. When compared against compounds in the ChEMBL_14, MDDR, Drug Bank and Dictionary of Natural Products, the percentage of virtual compounds with a Tanimoto index of 1.0 (ECFP_4) was found to be as high as 0.21. Depending on therapeutic target, this value may be in range of what might be expected from an experimental HTS campaign in terms of a true hit rate.

**Conclusion:**

Virtual compounds derived through enumerative combinatorics of simple atomic components have drug-like properties with ring assemblies that fall in new or under-represented areas of shape space. Structures derived in this manner could provide the starting point or inspiration for the design of structurally novel scaffolds in an unbiased fashion.

## Background

Contemporary small molecule drug discovery often relies on high-throughput screening (HTS) of either structurally diverse or mechanistically focused compound library sets to identify hits that have the potential for multiparameter optimization against biological targets of interest. Critical to the success of this approach is the availability of compounds in biologically relevant, druggable chemical space [1,2]. However, the concept of what constitutes a druggable chemical lead is evolving as more synthetically challenging molecules prepared through either semi- (e.g., Yondelis®, Ixempra®) or total synthesis (e.g., Halaven®, Aplidin®) pass through the clinical development pipeline to become marketed drugs. In recent years, for example, diverted total synthesis [3], diversity-oriented synthesis (DOS) [4] and biology-oriented synthetic (BIOS) [5] approaches have provided compounds for biological evaluation that possess increasing levels of structural and stereochemical complexity. Non-traditional lead-like molecules now include, for example, macrocyclic derivatives, which have been described in the literature as an underexploited structural class for drug discovery [6]. Finally, since natural products span regions of chemical space not represented by bioactive medicinal chemistry compounds [7], their scaffolds may serve as the inspiration for the design of structurally novel combinatorial libraries [8].

The goal of these efforts is to move into new or underexplored areas of chemical space with the expectation of finding activity against difficult to target protein systems with structurally novel compounds [9]. Since the pioneering work of Lipinski and co-workers [10], there has been an increased focus on bulk physicochemical properties of both leads and drug candidates as well as a better appreciation for drug space [11] in the context of the much larger chemical space [12]. Studies exploring the vastness of chemical space, for example, have led to the development of large virtual compound libraries through in silico combinatorial multiplexing [13,14]. While exhaustive libraries of this type have their place in virtual screening, the development of smaller dynamic virtual libraries focused on a particular mechanistic class may also contribute toward the identification of viable chemical leads.

Drug discovery, on the other hand, involves more than just interrogating proteins to identify small molecules that bind and invoke a functional response in vitro. It is typically a process of which hit identification marks just the beginning. The subsequent hit-to-lead evaluation and the success of lead optimization to identify a clinical candidate are critically dependent on the quality of the initial screening hit. In the extreme, if one limits the process to compound libraries with known activity and only screens against biological targets of known function using known methodology, then there is little need for innovation. Furthermore, past success may not necessarily predict future success [15,16]. Since complex interactions among protein systems may need to be modulated in the context of a disease state, new chemotypes may be required to enhance the arsenal of known scaffolds to explore new biological target engagement opportunities, and therefore drug discovery opportunities, in areas of unmet medical need.

While there are many complementary strategies to expand and enhance an existing screening library, one among several that we considered was the role of randomness in molecular assembly at the atomic level. We therefore decided to examine the importance of stochastic processes on molecular design, in particular, ring assemblies for identifying structurally novel biogenic-like scaffolds. Used in tandem with predictive in silico biological models as part of an iterative loop (i.e., enumerate, evaluate in silico, adjust enumeration conditions, repeat until convergence criteria satisfied), new structures could potentially be “evolved” in silico and used as the inspiration for structurally novel scaffold design. In this manner, virtual compounds could be generated in an unbiased fashion and filtered by in silico mechanistic models (e.g., kinases, GPCRs, PPIs, etc.) to create dynamic, structurally novel, mechanism-based screening libraries.

## Results

**Figure 1:**
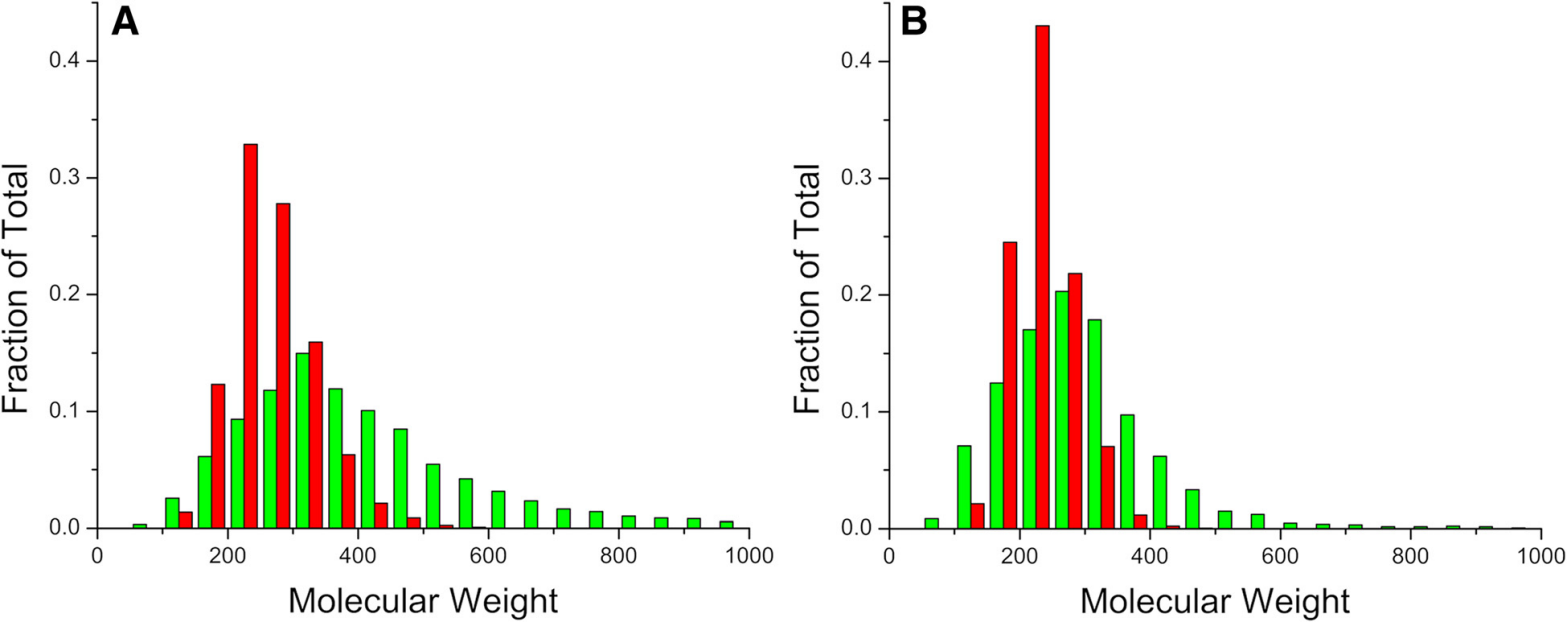
**MW histogram plots for the DNP (green) and enumerated (red) sets.** (**A**) Whole molecule comparison. (**B**) Ring assembly comparison.

In this study, known druggable space (KDS) was defined as the union of compounds found in the ChEMBL_14 [17], MDL/SYMYX Drug Data Report (MDDR release 2007.2) and DrugBank [18] databases with duplicate structures removed. Using a molecular enumerator developed in this laboratory [19], a total of 250,000 virtual compounds was generated from a carbon atom as the core with simple mono- and diatomic fragments added in a stochastic manner. As expected, analysis of the KDS, Dictionary of Natural Products (DNP) and enumerated sets indicate that the collection of randomly generated virtual compounds resembles that of the DNP with respect to the ratio of carbon, nitrogen and oxygen atoms present in each molecule, but with a narrower molecular weight range (Figure 1). This is a direct consequence of the larger number of oxygen containing fragments available for enumeration, where only one of the ten fragments contains a nitrogen atom.

**Figure 2:**
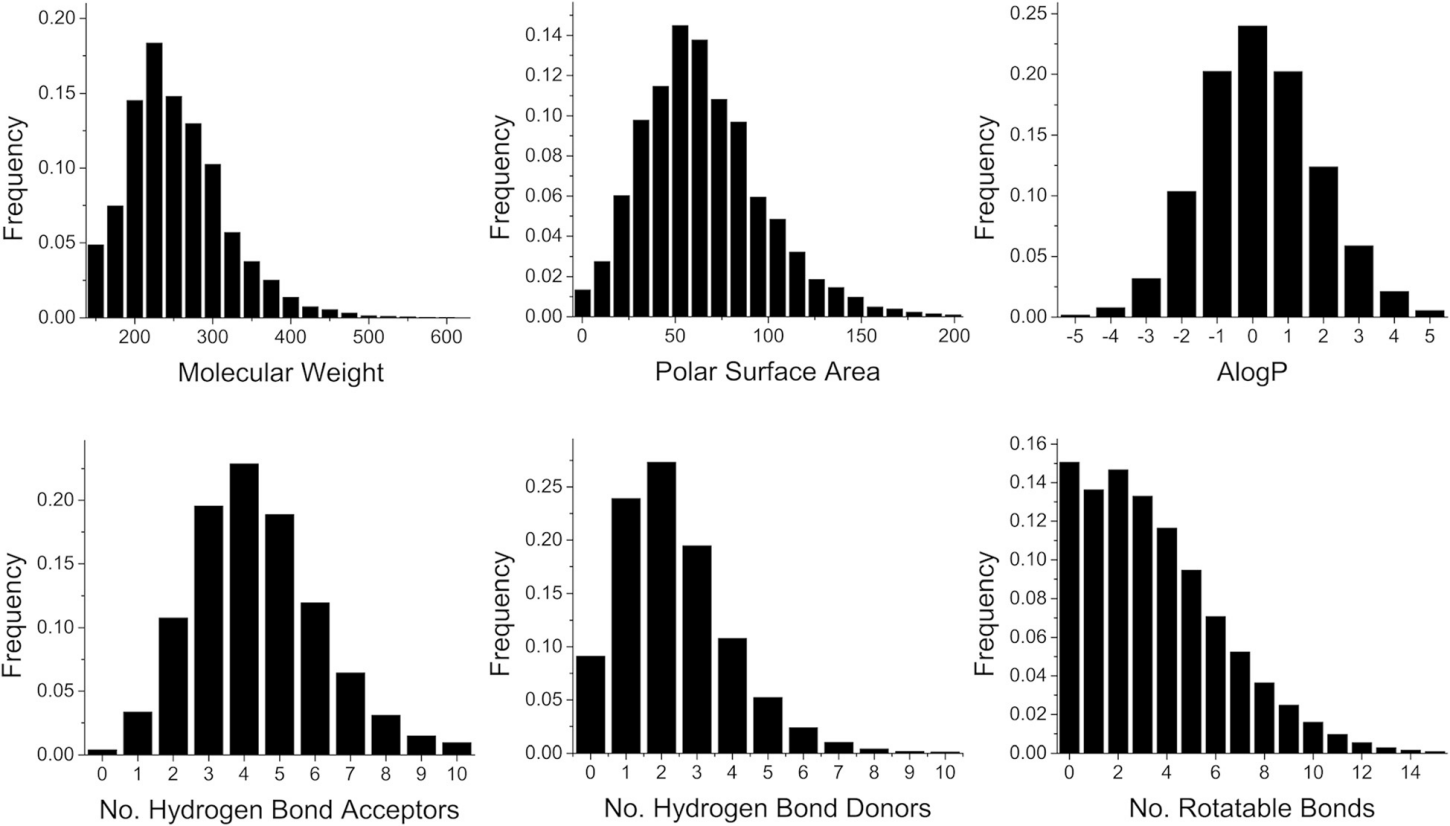
Histogram analysis of the 250,000 member enumerated virtual compound set.

**Figure 3:**
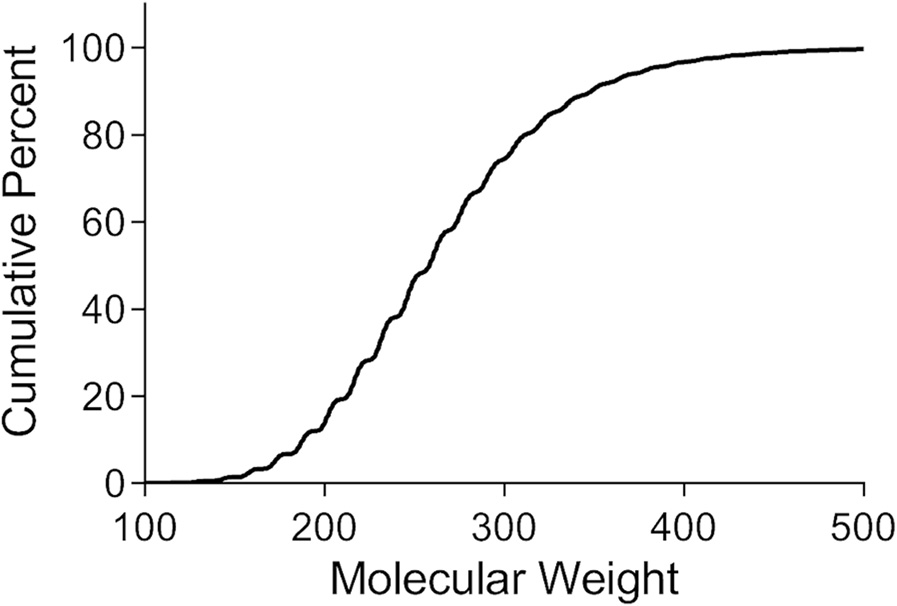
Cumulative plot of enumerated virtual compound numbers as a function of MW.

**Figure 4:**
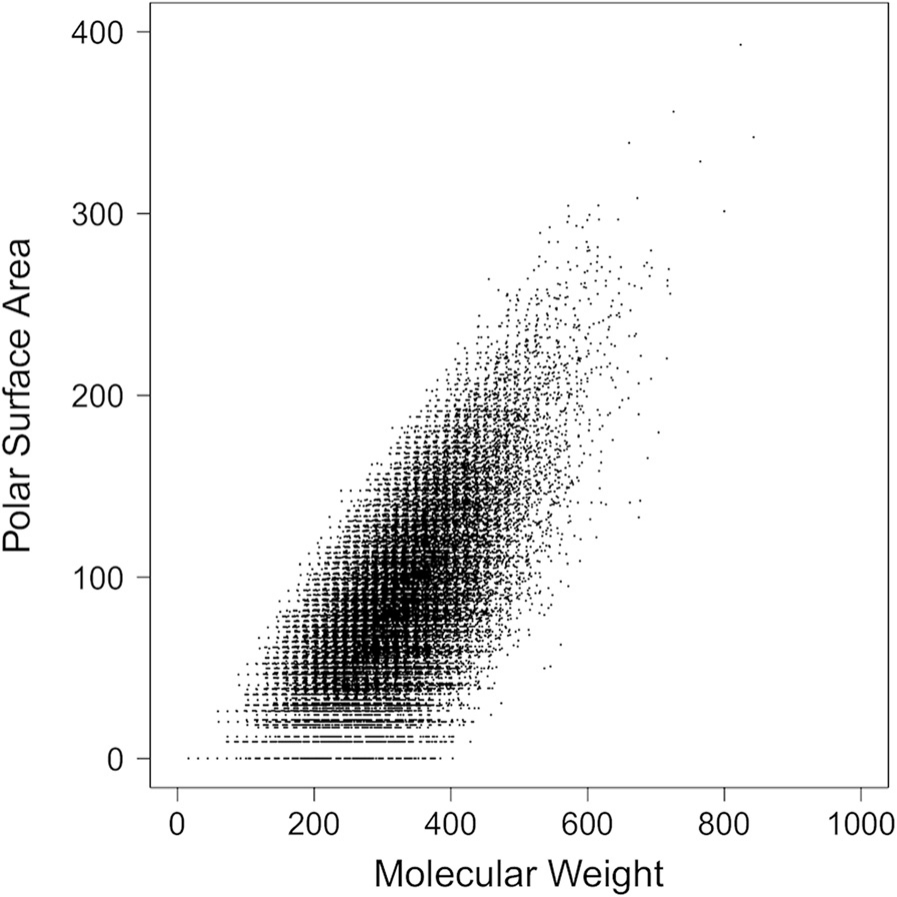
Plot of MW vs. polar surface area for the enumerated virtual compound set.

**Figure 5:**
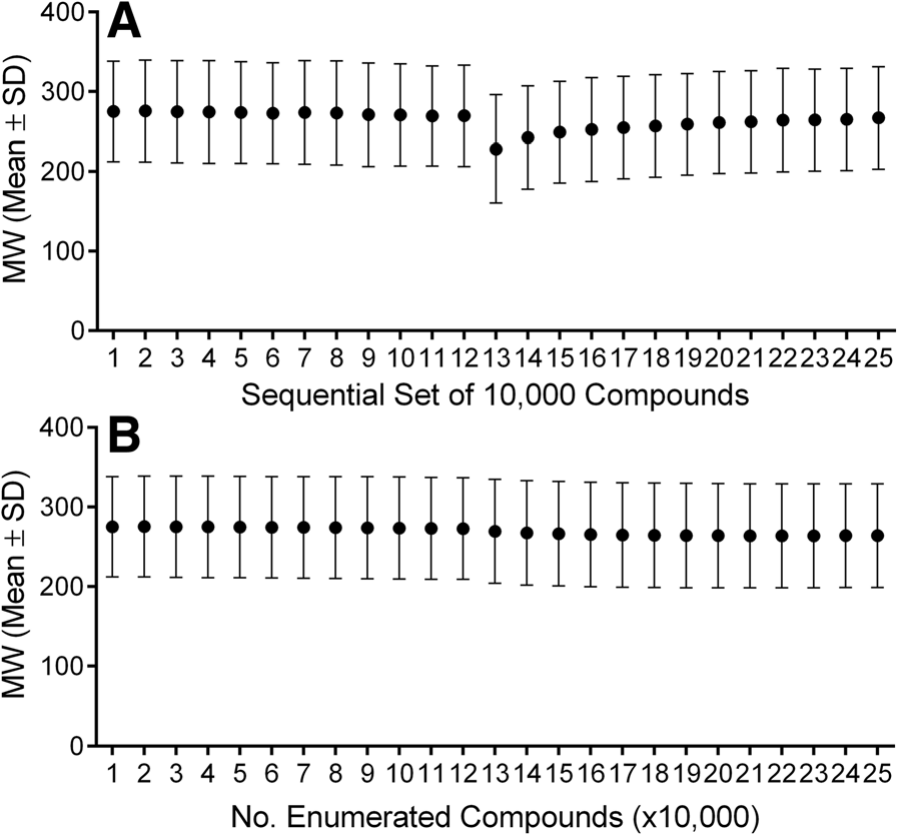
**MW plots of enumerated compounds (mean ± SD).** (**A**) For each sequential group of 10,000 enumerated compounds. (**B**) As a function of the number of compounds enumerated.

Interestingly, physicochemical property analysis reveals that a majority of the randomly generated virtual compounds falls in the range of generally accepted drug space (Figure 2), despite starting with a single carbon atom as the core and using only ten simple fragments. Here, the objective was not to explore all of possible chemical space, but to generate virtual compounds that could serve as the starting point or inspiration for designing structurally novel scaffolds. Of the enumerated structures, 99.6% have a MW less than 500 and 86% have a MW between 200 and 500 (Figure 3). By requiring the molecular polar surface area (PSA) to be greater than zero and less than 140 Å^2^, 83% of the enumerated structures fall in the MW range 200–500 (Figure 4). Furthermore, over 95% of the enumerated structures are both Lipinski [10] and Veber [20] rule compliant suggesting that a vast majority of the randomly generated virtual compounds possess physicochemical properties in the range appropriate for passive transcellular permeability with the possibility of oral absorption. Since each molecule grows in a stochastic and independent manner, the average molecular weight of the virtual set should not be dependent on the number of compounds enumerated. Monitoring the average molecular weight for each group of 10,000 compounds generated in sequence indicates no statistically significant differences between the groups (Figure 5A). Similarly, the average molecular weight over time does not change as the enumerated set grows from 10,000 to 250,000 members (Figure 5B).

**Figure 6:**
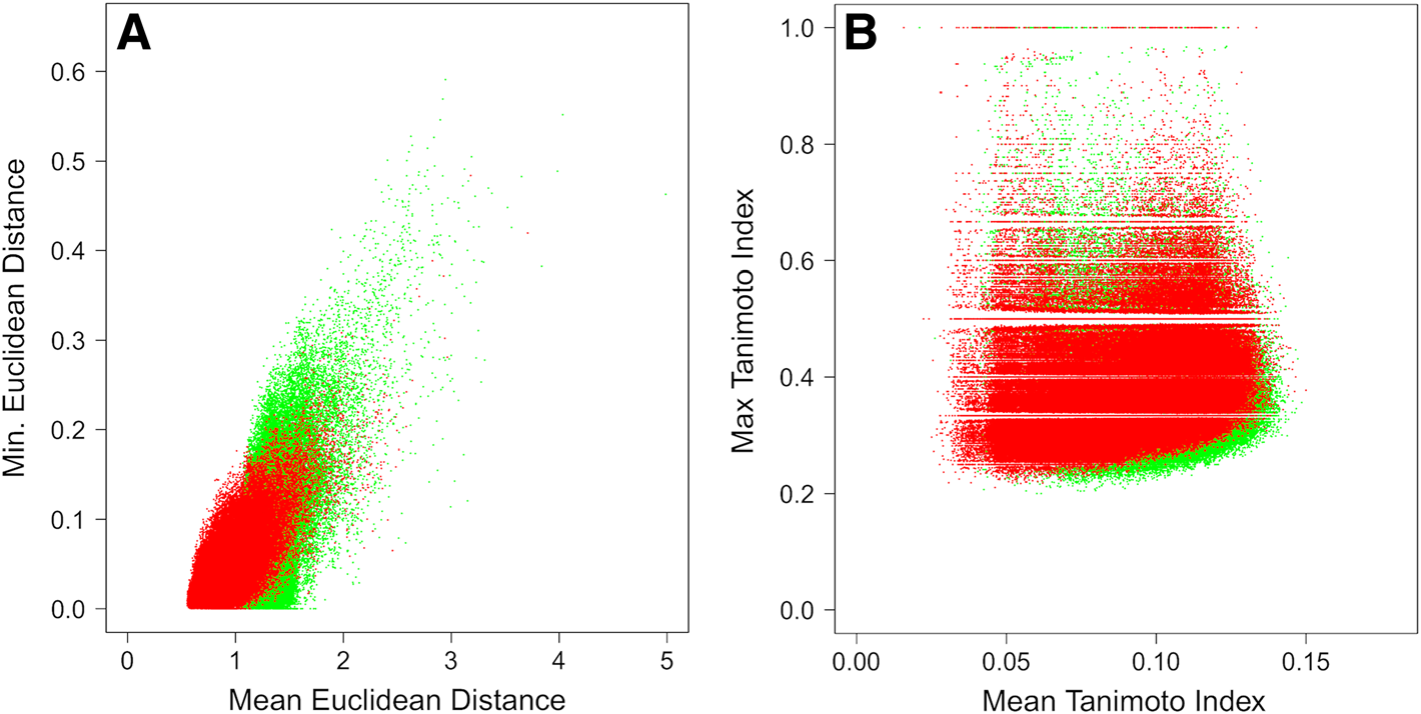
**Multi-fusion similarity maps plotting the virtual enumerated set against KDS (red) and DNP (green) as the reference sets.** (**A**) Euclidean distances using physicochemical properties MW, PSA, AlogP, HBA, HBD, RotB. (**B**) Tanimoto indices using ECFP_4.

Multi-fusion similarity analyses [21] of compounds in the KDS, DNP and enumerated sets further substantiate the conclusion that the virtual compounds resemble both of the former sets in terms of physicochemical properties (Figure 6A). Differences were noted, however, with respect to Tanimoto indices using ECFP_4 circular fingerprints (Figure 6B) [22]. Thus, the enumerated set differs structurally from both KDS and DNP compounds, but exhibit similar bulk physicochemical properties as evidenced by the average minimum Euclidean distance. These results suggest that the enumerated compounds are both structurally unique and drug-like in terms of their predicted properties. Inspection of Figure 6B reveals that a small percentage of the enumerated compounds exhibit a Tanimoto index of unity when compared against both the KDS and DNP sets. Of the 250,000 virtual compounds, 410 (0.16%) were found to meet this criterion.

To explore similarities that may exist in terms of molecular scaffolds, each of the three compound sets was reduced to a collection of ring assemblies using Pipeline Pilot [23] where original alpha atom attachments were maintained. In this manner, 95,522 different assemblies were identified from the enumerated set. Of these, 255 (0.27%) were found to be common with ring assemblies derived from either the KDS or the DNP sets.

**Figure 7:**
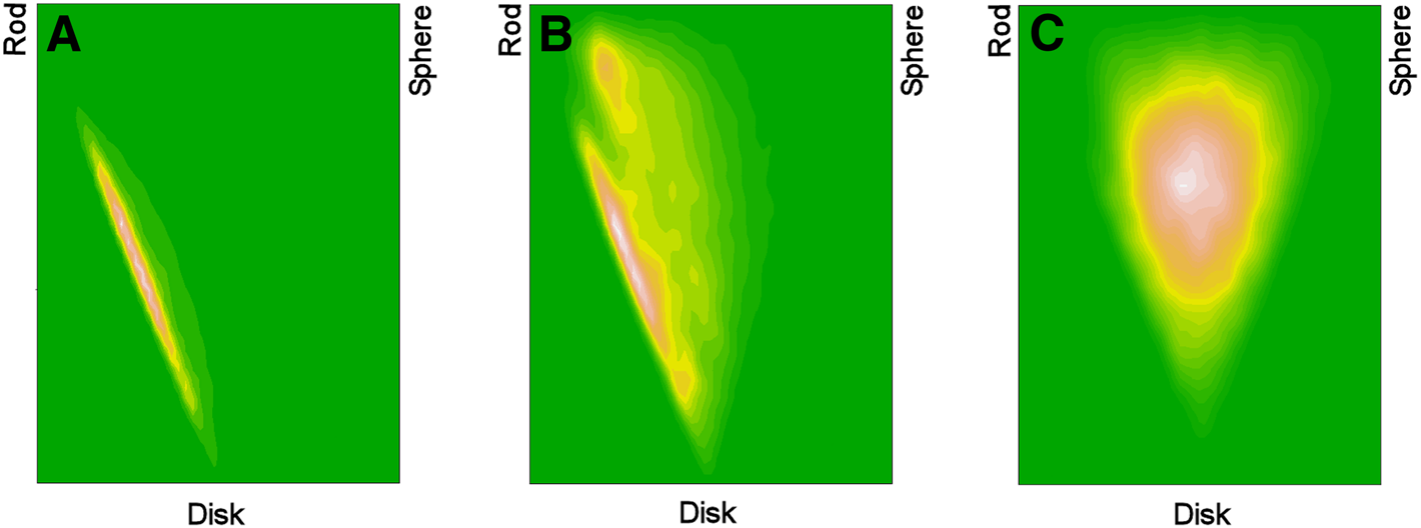
**PMI ring assembly shape distribution plot.** (**A**) KDS, (**B**) DNP and (**C**) enumerated set.

Using the principal moment of inertia (PMI) method of Sauer and Schwarz [24], considerable overlap was found to exist between the KDS and DNP ring assembly sets with respect to their 2D binned kernel density estimates. In contrast, the enumerated ring assemblies were found to occupy a much broader region centered in-between the rod, disc and sphere vertices of the shape space isosceles triangle (Figure 7).

To determine if the KDS and DNP ring assembly sets could be better differentiated from each other, an alternative method to assess and visualize molecular shape distribution in compound collections was investigated. Borrowing from the field of petrology, we explored a method originally developed to describe the shape of aggregates, material used to form the base coarse layer of roads [25]. In this approach, only the object’s degree of sphericity (ψ) and length along three major orthogonal axes would be needed. To accomplish this, we used shadow area and PMI as two different indices of molecular length. The elongation (p) and flatness (q) parameters, which are independent of molecular size, could then be calculated using equations 1 and 2, respectively. Molecular sphericity could be calculated using equation 3.

(1) $$p=\mathrm{small}\;\mathrm{value}/\mathrm{medium}\;\mathrm{value}\;\left(\mathrm{either}\;\mathrm{shadow}\;\mathrm{area}\;\mathrm{or}\;\mathrm{PMI}\right)$$

(2) $$q=\mathrm{medium}\;\mathrm{value}/\mathrm{large}\;\mathrm{value}\;\left(\mathrm{either}\;\mathrm{shadow}\;\mathrm{area}\;\mathrm{or}\;\mathrm{PMI}\right)$$

(3) $$\psi =\left(4\pi {\left(\sqrt[{3}]{\frac{3•\mathrm{SAVol}}{4\pi }}\right)}^{2}\right)/\mathrm{SASA}$$

Where:

SAVol = 3D surface area volume

SASA = 3D solvent accessible surface area

**Figure 8:**
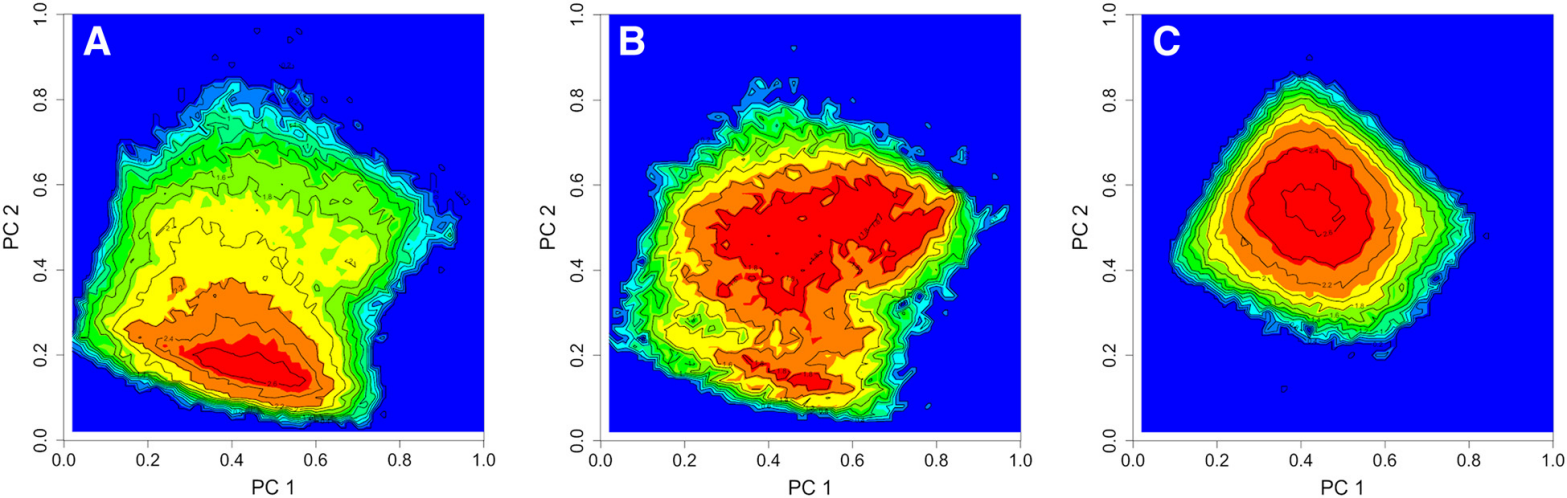
**Principal component ring assembly contour map using coarse aggregate shape descriptors p, q, and ψ.** The z-axis represented by the green to red color spectrum is the frequency of ring assemblies falling in that shape region. (**A**) KDS, (**B**) DNP and (**C**) enumerated set.

The KDS ring assembly set was used to build a PCA model using the five shape descriptors (p_shadow_area, p_PMI, q_shadow-area, q_PMI, and ψ) as independent parameters. The minimum and maximum values for the first two principal components calculated from the KDS and DNP sets were then used to scale the first two principal component values calculated for all of the ring assemblies. These were mapped onto a 50x50 grid, where the z-axis was taken to represent the frequency found in each square of the grid. A graph of these values revealed a better separation between ring assembly shapes found in the KDS and DNP sets (Figure 8). In this analysis, the randomly enumerated virtual compounds fall within known drug shape space, but with a density distribution more closely resembling that of the DNP.

**Figure 9:**
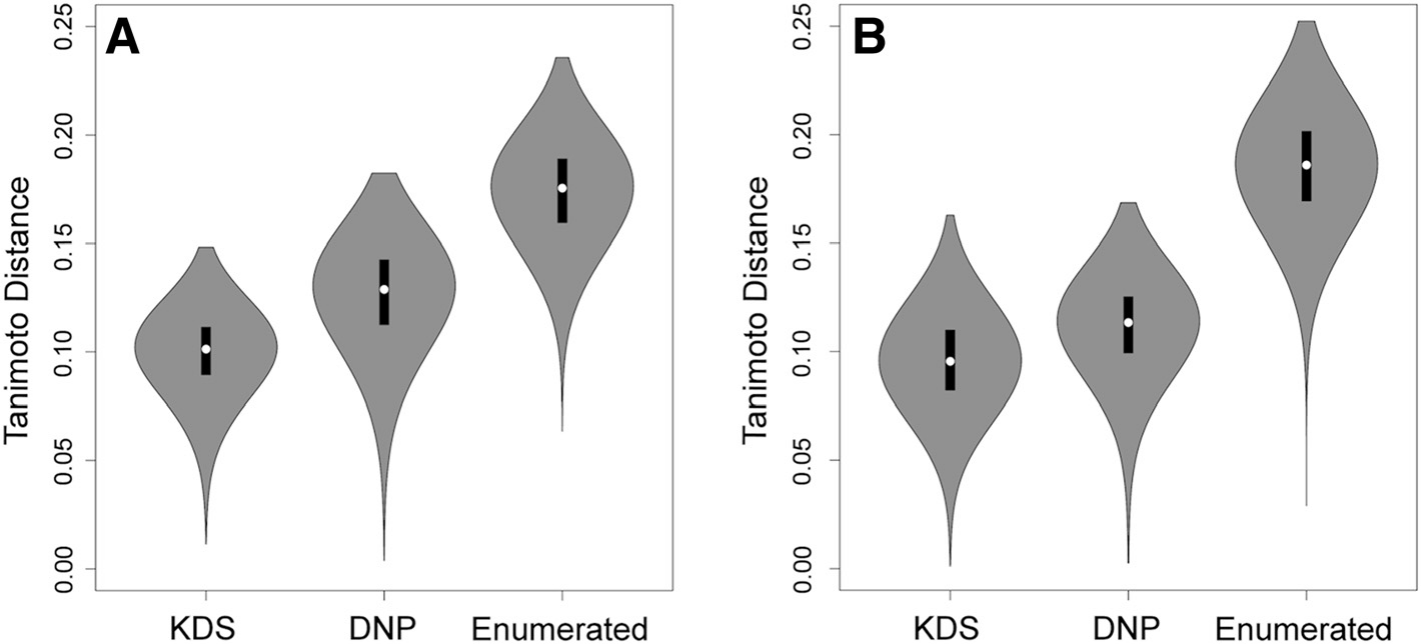
**Violin plots of the Tanimoto similarity distance using ECFP_4.** The distance was calculated for each ring assembly against all others in its respective set. (**A**) KDS, DNP and enumerated virtual compound ring assembly sets with original alpha atom attachments allowed. (**B**) Violin plot as before, but in the absence of alpha atoms.

**Figure 10:**
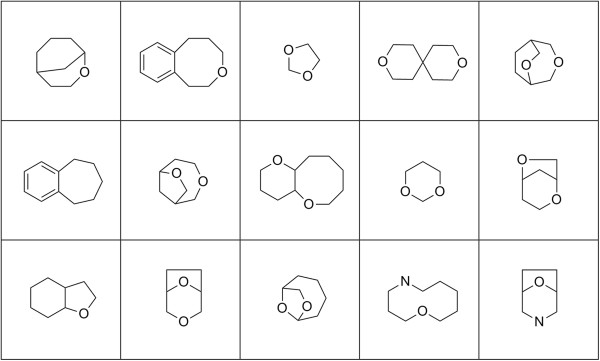
Sampling of ring assemblies without alpha atoms common to the enumerated set and either KDS or the DNP.

**Figure 11:**
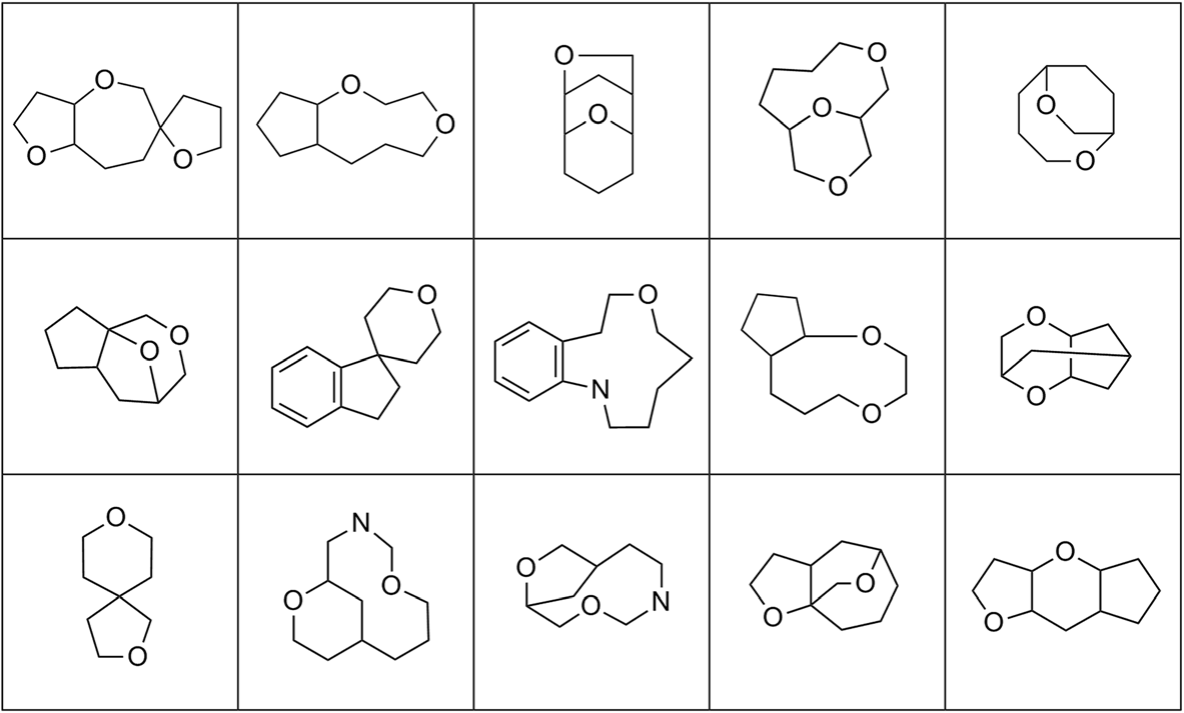
Sampling of structurally unique ring assemblies without alpha atoms from the complement of the enumerated set.

Scaffold diversity of the randomly enumerated compounds was assessed by calculating the Tanimoto distance (ECFP_4) for each ring assembly against all others in the set. For comparison, Tanimoto distances for the KDS and DNP sets were similarly calculated. While all three of the compound arrays were found to be internally structurally diverse with median Tanimoto distances less than 0.2, the enumerated set appears to be slightly less so than the other two (Figure 9A). This is likely a consequence of the enumeration fragments being limited to carbon, hydrogen, nitrogen and oxygen atoms with no halides, phosphorous, sulfur or other heteroatoms allowed. The ring assembly analysis was then repeated, but in the absence of alpha atoms (Figure 9B). The results were very similar, again indicating a high degree of internal structural diversity. A sampling of intersecting ring systems appears in Figure 10 and a sampling of unique ring systems from the complement of the enumerated set appears in Figure 11.

As expected, compounds in the enumerated virtual set exhibit a high degree of saturation with a mean ratio of sp^3^ hybridized carbons to total number of carbon atoms equal to 0.71 ± 0.22 (mean ± SD). In contrast, compounds in the KDS set exhibit a mean ratio of 0.34 ± 0.21. A similar difference was noted with their associated ring assemblies (original alpha atoms allowed), thereby suggesting that the enumerated scaffolds may possess an enhanced level of three-dimensional character relative to the KDS compounds. Since ring assemblies in the DNP exhibited a mean ratio of 0.61 ± 0.30, the enumerated compounds more closely resemble natural products in terms of their overall percentage of sp^3^ hybridized carbon atoms, which is to be expected given that all but two of the fragments available for enumeration are saturated.

To increase the amount of unsaturation in the enumerated structures, an olefin was added to the fragment set. A total of 250,000 virtual compounds was generated and analyzed as described earlier. Both the bulk physicochemical properties of the enumerated molecules and the shape distribution of the corresponding ring assemblies were found to be very similar (see Additional file 1). Thus, adding unsaturation in the form of non-aromatic double bonds did not alter either the calculated bulk physicochemical properties of the virtual compounds or the shape of the corresponding ring assemblies.

**Figure 12:**
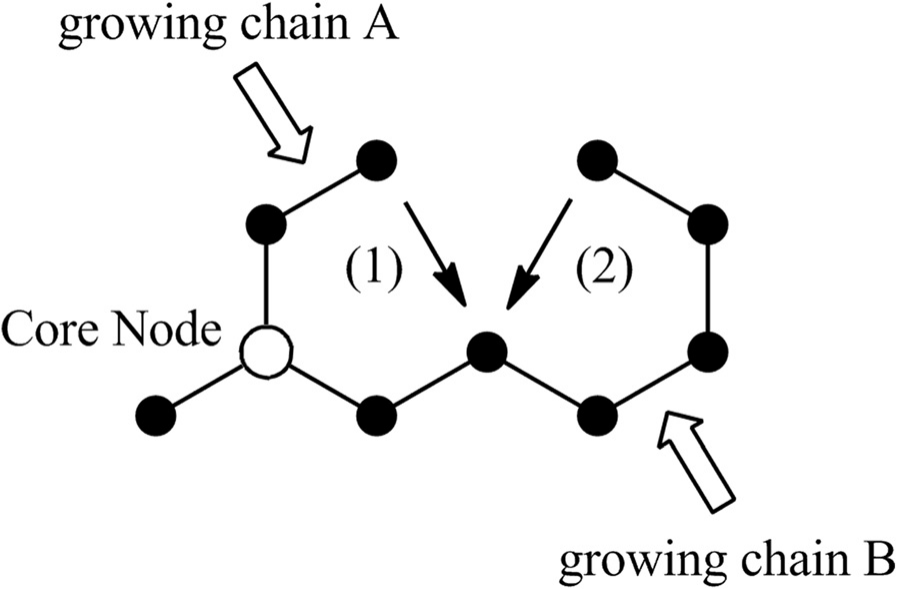
**Cartoon depicting two different ring forming modes used by the enumerator.** (1) Inter-chain ring formation between growing chains A and B. (2) Intra-chain ring formation within growing chain B.

The effect of allowable ring formation modes was then investigated with respect to the type of compounds generated (Figure 12). In these runs either the intra-chain mode of ring formation or the inter-chain mode was deactivated. The bulk physicochemical properties for the two modified enumerations were again found to be very similar indicating that the ring forming mode did not impact the calculated properties of the virtual compounds (see Additional file 1). Allowing both ring forming modes to take place afforded structures of which 95% were both Lipinski and Veber rule compliant. Similarly, allowing only the intra-chain or the inter-chain modes to occur in separate runs gave structures that were 86% and 96% compliant, respectively. When compared against the KDS and DNP sets using ECFP_4 fingerprints, the 250,000 virtual compounds with the intra-chain option deactivated afforded 270 virtual “hits” (0.11%) with a Tanimoto index of 1.0. For the set with the inter-chain option deactivated, a total of 521 “hits” (0.21%) emerged.

**Figure 13:**
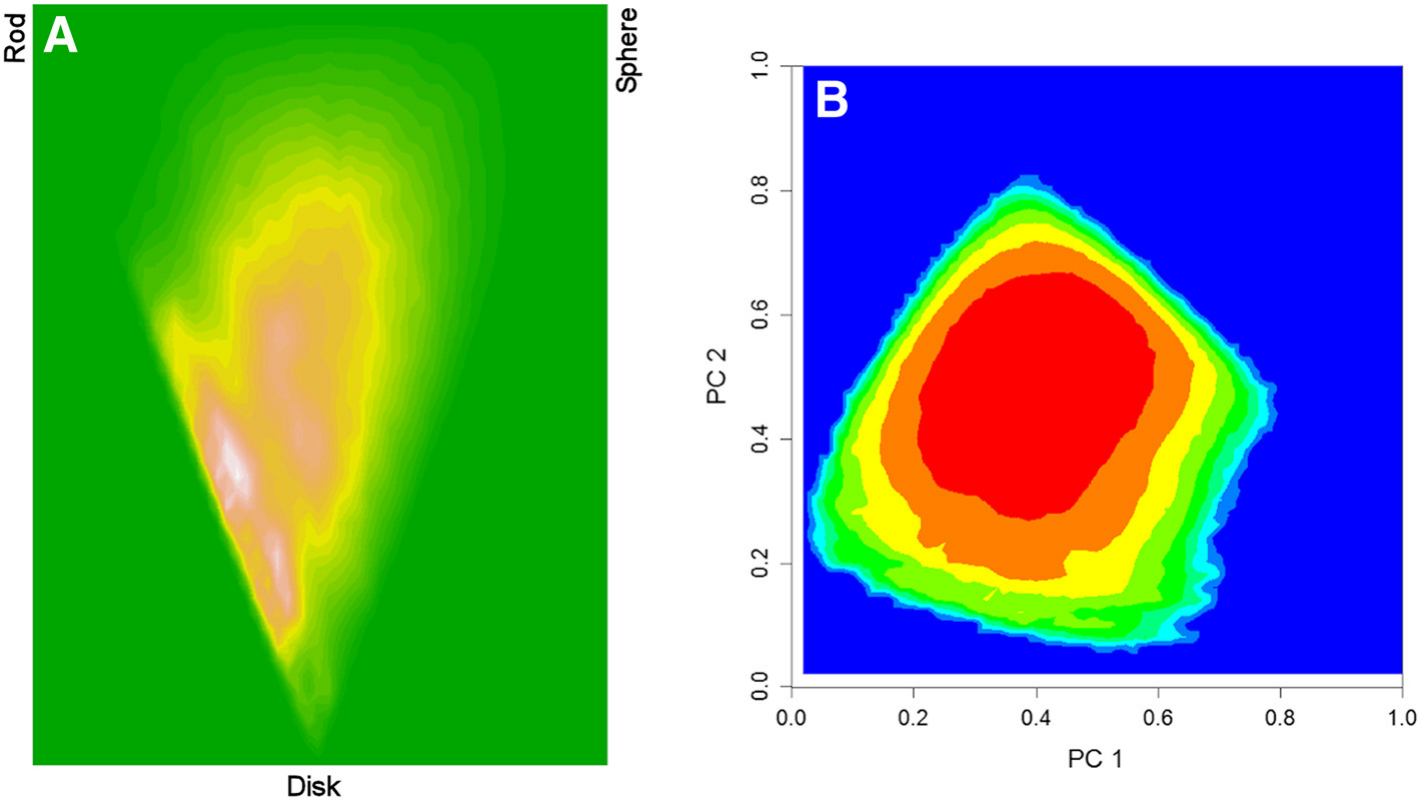
**Shape distribution plots for random 1 million compounds from GDB13.** (**A**) PMI ring assembly shape analysis. (**B**) Principal component ring assembly shape analysis.

For comparison, the random 1 million compound set from the GDB13 database [14] was downloaded [26] and analyzed. The average molecular weight for this set was 178 ± 10 (mean ± SD), whereas for the enumerated set of 250,000 compounds the average molecular weight was 264 ± 65. Other than the difference in average molecular weight, the distribution of total polar surface area, AlogP, number of rotatable bonds, number of hydrogen bond donors and number of hydrogen bond acceptors was similar across the two sets (see Additional file 1). With regard to molecular shape, the ring assemblies associated with the random 1 million GDB13 structures span the region occupied by both the enumerated and DNP ring assemblies (Figure 13).

**Figure 14:**
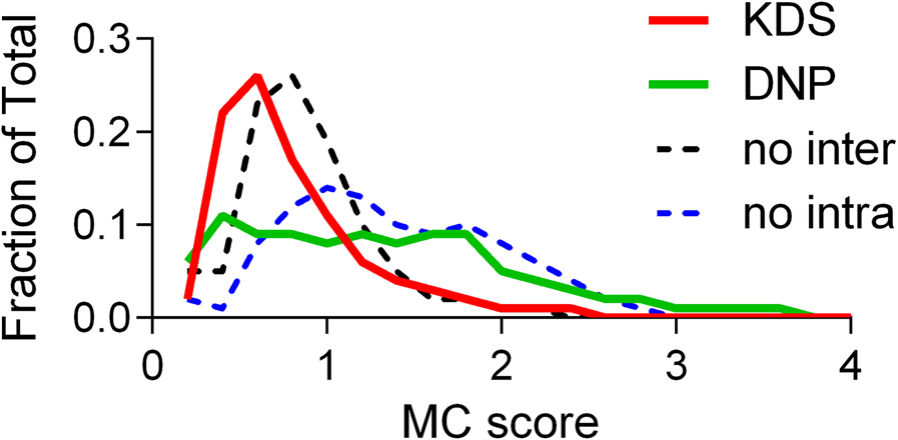
Molecular complexity (MC) score distribution for the KDS, DNP and enumerated sets.

The enumerated and GDB13 sets were then scored by molecular complexity (MC) as calculated using an adaptation of the method described by Ertl and Schuffenhauer [27]. The resulting distribution was compared to that found for the KDS and DNP sets. Inspection of Figure 14 indicates that disabling the inter-chain ring forming mode afforded virtual compounds calculated to be more like those in the KDS in terms of their MC score, whereas disabling the intra-chain mode generated structures that more closely resembled those of the DNP.

$$\begin{array}{c} \mathrm{Molecular}\;\mathrm{Complexity}\;\left(\mathrm{MC}\right){\;\mathrm{score}=\log}_{10}\left(\mathrm{nBridgeAtoms}+1\right){+\log}_{10}\left(\mathrm{nSpiroAtoms}+1\right)\\ \;+\log_{10}\left(\mathrm{nStereoCenters}+1\right){+\log}_{10}\left(\mathrm{nMidLargeRings}+1\right)\\ \;{+\log}_{10}\left(\mathrm{nNonAromaticDoubleBonds}+1\right)+{\left(\mathrm{nHeavyAtoms}\right)}^{1.005}\\ \;-\mathrm{nHeavyAtoms} \end{array}$$

**Figure 15:**
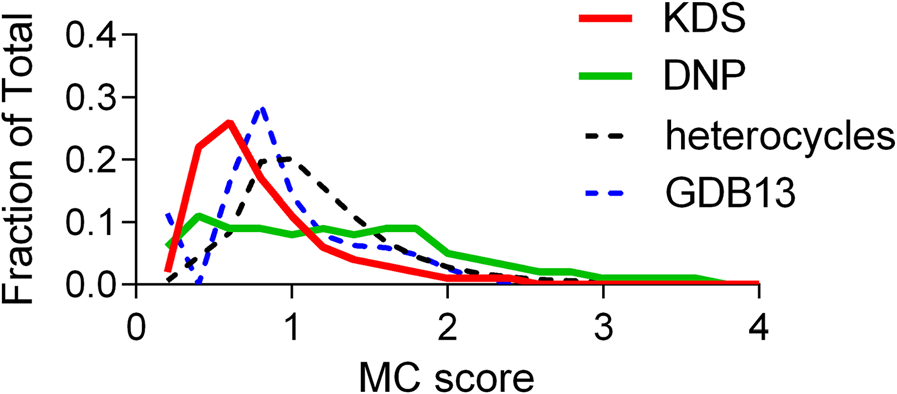
Molecular complexity score (MC) distribution for the KDS, DNP, heterocyclic enumerated and GDB13 sets.

A number of different heteroaromatic ring systems (pyridine, pyrimidine, indole, benzimidazole, purine, etc.) were added to the fragment pool to allow these ring systems to be incorporated into the enumerated structures. In this case, of the 250,000 virtual compounds 62% were both Lipinski and Veber rule compliant despite an increase in the average molecular weight of the set as a whole (467 ± 194, mean ± SD). Thus, even with an expanded fragment collection more than half of the enumerated compounds fall within bulk physicochemical space generally associated with orally active drugs. As expected, the molecular complexity score for this group of virtual compounds falls in-between that of the KDS and DNP sets, and is similar to that of the random 1 million compound GDB13 set (Figure 15).

## Experimental

The KDS and DNP compounds were deglycosylated using Pipeline Pilot prior to analysis. The enumerated sets were generated as follows: A total of 250,000 virtual compounds consisting of both acyclic and cyclic systems was randomly generated from a single carbon atom in the absence of any molecular weight (MW) constraints (either minimum or maximum). The fragments appear in Table 1. Fragment weights were scaled to according to the number of carbon atoms found in the largest fragment.

**Table 1:** **Fragments used in the enumeration process**[19]

**Number**	**Fragment**	**Allow heteroatom attachment**	**Enumeration weight**
1	H	yes	6
2	*C^P^	yes	6
3	*C^P,D,A^	yes	6
4	*N^P^	no	6
5	*O^T^	no	6
6	*O^P^	no	6
7	^*^O^D^	NA	6
8	^P^*C-O	no	6
9	^A^*C = O	yes	6
10	Phenyl (*C1,C2^D^)	yes	1
11	*C = C^P^	no	3

Note that a single atom can serve more than one function. It can be (1) the point of initial attachment in a growing chain, (2) the point of propagation, (3) a donor or acceptor ring forming node.

NA: Not applicable.

*Location of initial attachment to growing chain.

^T^Terminating node.

^P^Propagating node.

^D^Ring forming donor node.

^A^Ring forming acceptor node.

## Discussion

Appropriate physicochemical properties are important parameters for compounds to become drugs; they affect the route of administration, pharmacokinetics, pharmacodynamics, formulation and chemical stability of the drug substance. Thus, it is critical during lead optimization that these properties be either retained or optimized so that the compound reaches its biological target with sufficient exposure to elicit a functional response in vivo with an acceptable side effect profile. Starting with a lead compound that already falls in accepted physicochemical drug space will likely streamline the path to both in vivo proof of concept and eventually clinical candidate selection. For example, optimizing for CNS penetration involves multiple parameters that include physicochemical properties [28].

The work by Lipinski and co-workers reinforced the importance of physicochemical property space with regard to oral drugs, which served to highlight that intrinsic potency is just one of many parameters that must be considered during lead optimization. Yet, potency is an important factor that drives the choice of analogues to be progressed based on indices such as ligand efficiency [29]. As pioneered by Paul Ehrlich in the early 20^th^ century, compounds must interact with a biological target to elicit a biological response. This interaction can be explained by conventional chemical interactions and, in a deterministic sense, reduced to a set of atomic features consisting of electrostatics, hydrogen bonds, van der Waals, hydrophobic and pi stacking interactions as well as entropic considerations. Since biological targets (e.g., protein receptors, enzymes and DNA) are three-dimensional structures, the interactions require appropriate complementary placement of the pharmacophoric groups in three-dimensional space. Thus, biologically active molecules arise as a consequence of appropriate pharmacophoric feature pairing with their biological target(s). As a result, adequate representation in shape space in addition to property space will be important for a screening library to successfully provide viable chemical leads for optimization and in vivo proof of concept.

In this regard, the majority of natural product scaffolds have been shown to be absent from commercially available compounds [30], consistent with the shape analyses depicted in Figures 7 and 8. The directed expansion and enhancement of existing HTS screening libraries for drug discovery purposes could therefore be accomplished not only through the addition of compounds biased toward naturally occurring biogenic scaffolds (e.g., DOS or BIOS), but also through the addition of biogenic-like scaffolds derived from enumerative combinatorics of simple atomic components. When compared with compounds from the KDS and DNP sets, the enumerated compounds exhibited a virtual “hit” rate as high as 0.21% (i.e., Tanimoto index = 1.0). Depending on therapeutic target, this value may be similar to what might be expected from an experimental HTS campaign in terms of a true hit rate.

The question then arises as to whether or not the collection of molecular shapes associated with known small molecule ligands will be sufficient to support future drug discovery efforts that will likely include modulating therapeutic targets for which there are currently no known small molecule effectors. Since biologically active synthetic compounds and natural products work with known systems, it logically follows that screening libraries could be enhanced and expanded based on the concept of privileged structures [31]. If, however, a subset of future therapeutic targets requires ligands that possess molecular shapes not represented by known drugs, then enhancing screening libraries with compounds derived through enumerative combinatorics may be appropriate. In this sense, the results of this study do not contradict the concept of DOS and BIOS [32], but rather complements it in terms of potential future unknown therapeutic target opportunities. Since the enumerator does not utilize chemical reaction information in the construction of virtual compounds, the resulting structures may point the way to new scaffolds in unexplored areas of molecular shape space. Thus, this approach may be complementary to those that employ known chemical reactions and common molecular building blocks to generate virtual compound libraries [33].

Since the molecular mass distribution of drugs is essentially identical to that of all chemicals stored in the Beilstein database, it’s tempting to speculate that randomness may indeed play a role in drug design, i.e., that drugs arise from a random sampling of existing substances defined by the rules of organic chemistry as proposed by Fialkowski and co-workers [34]. Alternatively, the overlapping molecular weight distribution of random molecules and drug substances could be viewed as additional evidence that physicochemical space is the consequence of a stochastic process that is independent of synthetic pathways.

During drug discovery, compounds that are found to exhibit desirable biological activity are expanded upon during the hit to lead and lead optimization phases, while those that do not are either discarded or remain as singletons. Since these analogues are usually retained in the screening library, over time the collection can become enriched in chemotypes associated with past programs, which may limit the probability of success with therapeutic targets outside historical mechanistic classes. In such cases, enumerative combinatorics in combination with in silico biological models may represent one approach to enhance and expand existing screening libraries in an unbiased fashion.

## Conclusion

This study suggests that bulk physicochemical property drug space could have arisen from enumerative combinatorics independent of synthetic pathway. Since natural products are produced by organisms through biosynthetic pathways and non-naturally occurring drug substances are generally produced through chemical reactions performed in the laboratory using different techniques and starting materials, it might be expected that the two classes of compounds differ from each other and can, in fact, be distinguished on the basis of chemical fingerprints (e.g., HOSE codes) [35]. The enumerated molecules, however, were constructed in a stochastic manner following neither paradigm and yet are predicted to exhibit bulk physicochemical properties consistent with known biologically active agents, whether they be naturally occurring or not. Since these structures fall in new or under-represented areas of shape space, virtual compounds derived through enumerative combinatorics of simple atomic components could provide the starting point or inspiration for the design of structurally novel scaffolds in an unbiased fashion that blur the line between synthetic substances and natural products.

## Supplementary Material

Additional file 1 **Figure S1.** Histogram analysis of enumerated structures with an olefin fragment included (see Experimental Section, Table 1 of the manuscript for details). **Figure S2.** Histogram analysis of the GDB13 set (1 million random compounds) as downloaded from the website. Click here for file
