# Supplementary material for: Druggable chemical space and enumerative combinatorics
Source: J Cheminform. 2013 Apr 18;5:19. doi: 10.1186/1758-2946-5-19 (PMC3641967; doi:10.1186/1758-2946-5-19)
Supplement: Additional file 1 — Figure S1. Histogram analysis of enumerated structures with an olefin fragment included (see Experimental Section, Table 1 of the manuscript for details). Figure S2. Histogram analysis of the GDB13 set (1 million random compounds) as downloaded from the website. [file 1758-2946-5-19-S1.pdf]

Supplementary Material

Druggable Chemical Space and Enumerative Combinatorics

Melvin J. Yu

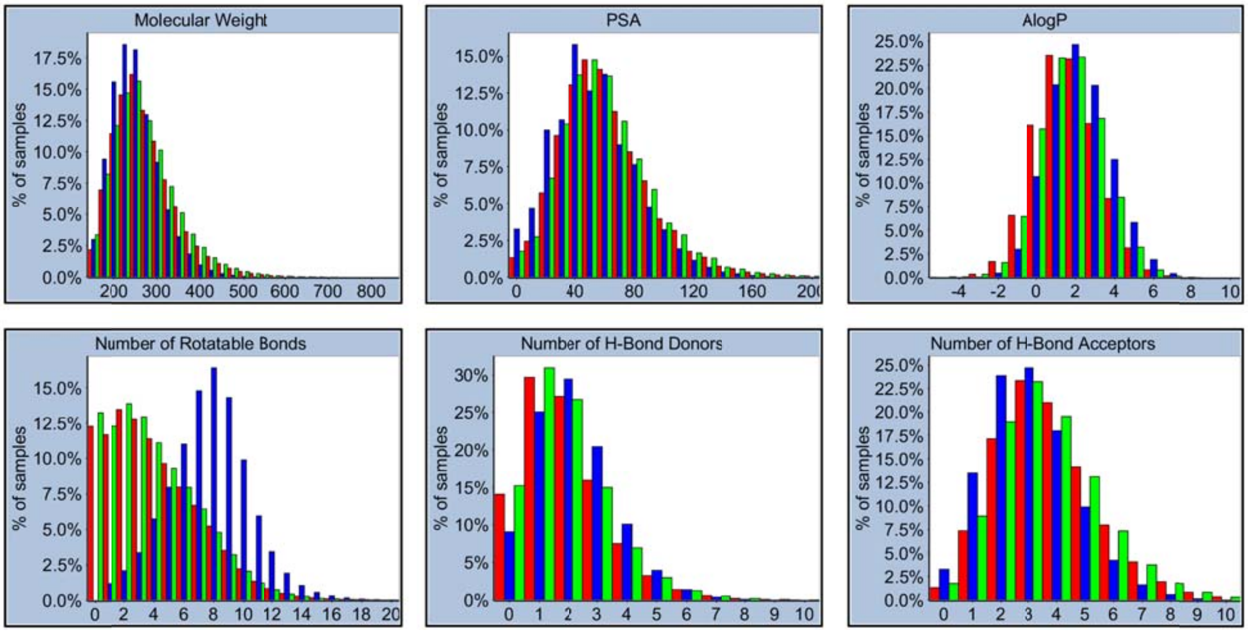

Supplemental Figure 1. Histogram analysis of enumerated structures with an olefin fragment included (see Experimental Section, Table 1 of the manuscript for details).

Each set consists of 250,000 virtual structures. One set was enumerated with the intra-ring formation option disabled (green). Another set was enumerated with the inter-ring formation option disabled (blue). The last set had neither disabled (red).

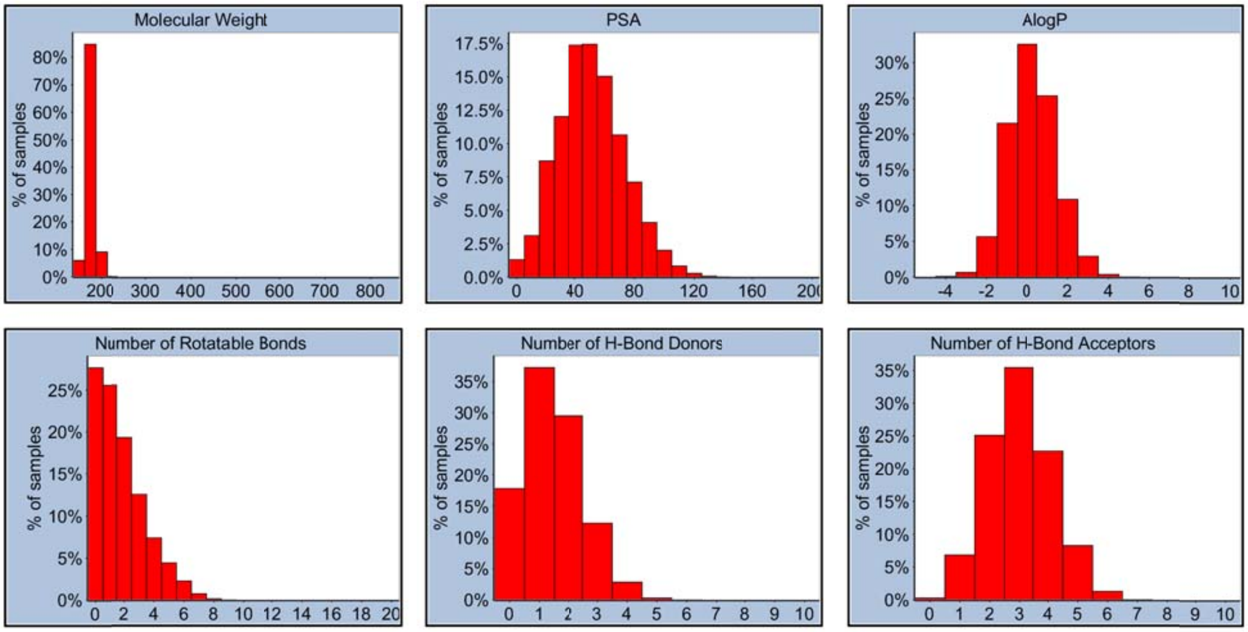

Supplemental Figure 2. Histogram analysis of the GDB13 set (1 million random compounds) as downloaded from the website.
